# PREDICTIONS FOR DEMENTIA BURDEN AND POLICY RESPONSES IN CHINA AND UK BY 2050

**DOI:** 10.1093/geroni/igad104.0645

**Published:** 2023-12-21

**Authors:** Jing Liao, Eric Brunner

## Abstract

The goal of this symposium is to provide a forum to interact with the principal communities, who are developing research on the projection of dementia burden to ageing society and policy responses. It is driven by the UK-China Health and Social Challenges Ageing Project (UKCHASCAP). China’s rapid ageing process is occurring at an earlier stage of economic development than many other countries including UK, posing great demands on families, communities and health and care services. Thoughtful comparison of health and social care policy across the contrasting countries will be scientifically illuminating and valuable to policy makers. Preliminary results from four studies will be presented. Two studies based on large ageing cohorts of China and the UK, using multi-state Markov modelling (IMPACT-CAM, IMPACT-BAM) to construct predictions of dementia and related health status (e.g. CVD and physical disability) to 2050. The British model will highlight calculation of dementia incidence trend over time. The following study further used IMPACT-CAM to predict socioeconomic cost of medical, informal and formal care, and quality adjusted life years associated with dementia in China in corresponding years. The fourth study systematically evaluates the effects of implementing long-term care insurance in pilot cities in China, and analyses the main challenges in establishing a nationwide equitable long-term care system for older people. Through this symposium, the panel will provide state-of-art modelling and latest data on the innovative, interdisciplinary and international collaboration for predicting dementia, and provide scientific advices for policy making on healthy ageing in China, the UK and globally.

